# Arc Expression in the Dentate Gyrus of F344 Rats Across the Sleep-Wake Cycle

**DOI:** 10.17912/micropub.biology.002282

**Published:** 2026-08-14

**Authors:** Zaira J. Rehman, Andres J. Socarras, Joshua G. Player, Craig Myrum

**Affiliations:** 1 Department of Biology, Loyola University Maryland, Baltimore, MD, United States

## Abstract

The Arc gene is rapidly expressed in the dentate gyrus of the hippocampus during learning and is essential for long-term memory formation. Although memory consolidation, which occurs predominantly during sleep, depends on Arc, previous studies have reported conflicting findings regarding whether Arc expression changes across the sleep-wake cycle. As an initial step toward addressing these discrepancies, we established a baseline comparison of Arc expression in the dentate gyrus of naïve Fischer (CDF) rats (n = 44) across four timepoints spanning sleep and wake periods. Arc⁺ cell density remained remarkably stable across all timepoints.

**Figure 1. Arc cell density in the dentate gyrus across the sleep-wake cycle:** ( **A** ) Percentage of total time spent sleeping during the three hours prior to sacrifice, confirming that each animal was largely sleeping or awake during the light or dark periods, respectively. ( **B** ) Representative Arc immunohistochemistry of a whole hippocampus section, along with a magnified image of the dentate gyrus. ( **C** ) Arc⁺ cell density in the DG during four timepoints across the sleep-wake cycle.

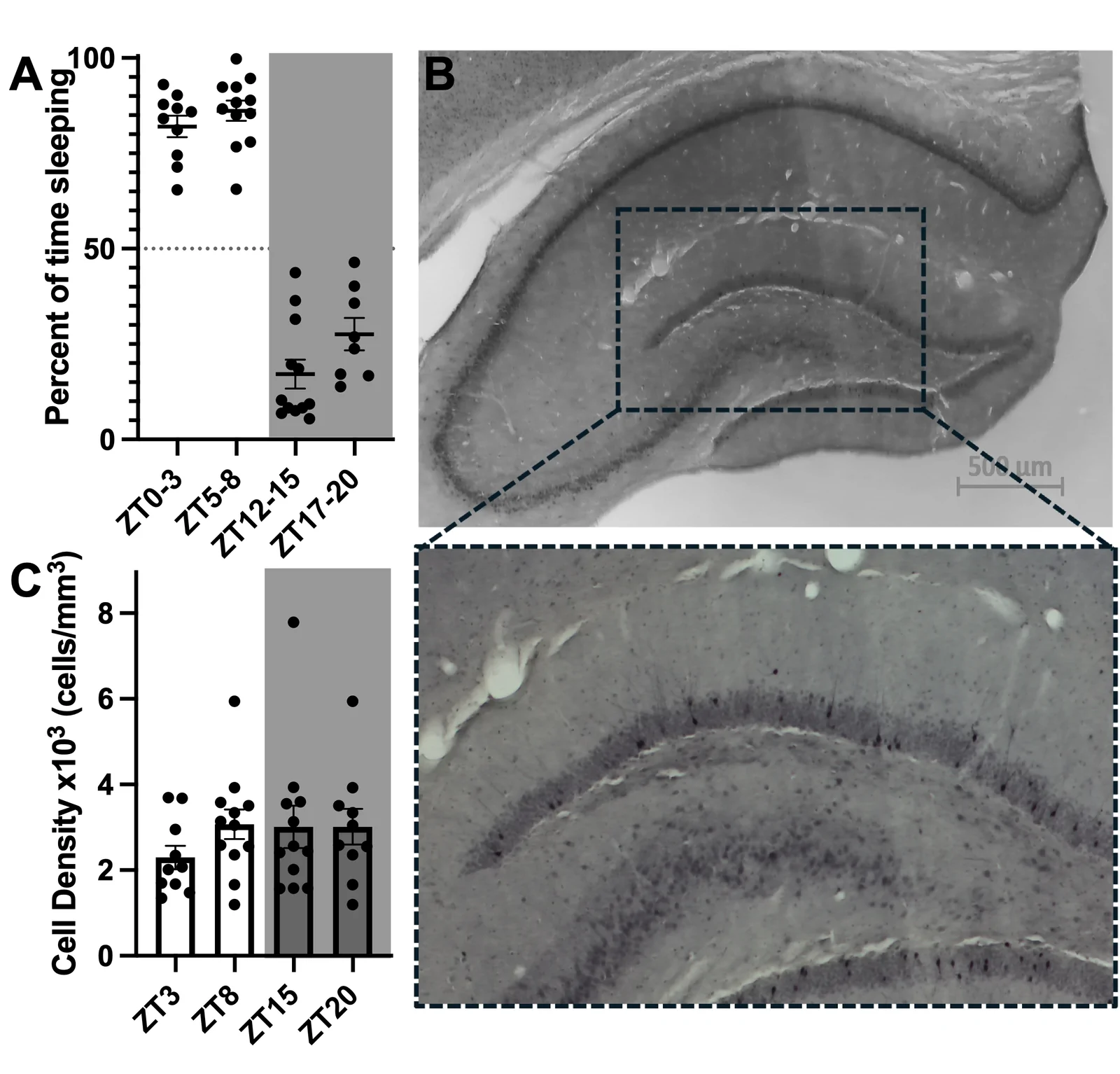



## Description

Sleep plays a fundamental role in memory consolidation by facilitating the stabilization of newly encoded information and its subsequent reorganization into long-term memory representations. Specifically, coordinated neural activity—including hippocampal–cortical oscillations—supports the reactivation of learning‑related neuronal ensembles and their integration with distributed cortical networks (Rasch & Born, 2013). This reactivation is thought to bias synaptic plasticity toward recently encoded experiences, strengthening relevant connections while weakening competing representations (Ólafsdóttir et al., 2018). Together, these processes promote both synapse‑level refinement and systems‑level reorganization of memory traces.

Among the many genes underlying the plastic processes that support memory consolidation, activity‑regulated cytoskeleton‑associated protein (Arc) stands out as a “master regulator” by virtue of its ability to mediate both synaptic strengthening and weakening (Myrum et al., 2022; Sullivan et al., 2025). Arc functions as an interactive molecular hub, associating with 30+ binding partners, enabling it to coordinate multiple intracellular signaling cascades and forms of plasticity (Nikolaienko et al., 2018; Zhang & Bramham, 2021). Arc is rapidly induced by neuronal activity associated with experience such as learning, highlighting its functional role as a molecule that acts within defined neuronal populations to shape circuit plasticity. Following spatial exploration, Arc protein is rapidly yet sparsely expressed in dentate gyrus (DG) granule cells (Ramírez-Amaya et al., 2005)—a hippocampal region that serves as the principal cortical input to the hippocampus and is critically involved in pattern separation. Given substantial functional and physiological differences in Arc expression between regions, the current study specifically examined the DG subregion. Arc expression in the DG is highly restricted to a small population of strongly activated neurons, unlike the more widespread recruitment seen in other hippocampal and neocortical areas (Ramírez-Amaya et al., 2005; Vazdarjanova et al., 2006), and is consistent with sparse neuronal recruitment during pattern separation. Following neural activity, Arc expression is sustained for hours in DG granule cells but is transient in pyramidal cells, decaying within ~1 h after learning or long-term potentiation (Zhang & Bramham, 2021).

Since sleep is a critical period for memory consolidation and this process depends on Arc function, it follows that Arc levels may be increased during sleep. However, there is little agreement as to whether—and in what direction or in which brain region(s)—Arc levels change during sleep. Given the hippocampal subregion specificity of REM-induced Arc expression (Renouard et al., 2015), there is reason to suspect that different hippocampal and neocortical regions may display different patterns of basal Arc expression. Indeed, earlier studies have shown that in some brain regions, Arc expression is higher during wake than sleep. For example, microarray data showed that cortical Arc mRNA levels during sleep were roughly half of that observed during wakefulness, and the number of Arc immunopositive cells in the parietal cortex during sleep was approximately one-third of wake levels (Cirelli & Tononi, 2000). In contrast, in whole mouse hippocampi and neocortex, proteomic analyses showed no significant differences in Arc levels when comparing ZT6 and ZT18 (i.e. at the midpoint of the light and dark periods, respectively) (Bering et al., 2023). In another study, post-learning sleep was associated with decreases in Arc gene expression across 2-6 h of sleep relative to wake home cage controls—an effect seen in the hippocampus and especially robust in the cortex (Genzel et al., 2017). Perhaps the best evidence for diverging patterns of sleep-associated Arc induction comes from a study in male Sprague-Dawley rats, where Arc transcript levels increased during the dark/active period in the adult neocortex, while hippocampal levels remained stable across 24 h (Bille et al., 2026). Another study reporting regional specificity showed that sleep deprivation in mice simultaneously decreased Arc⁺ cells in the DG, and increased Arc⁺ cells in the cortex (Delorme et al., 2018).

As an initial step to address discrepancies between earlier studies, we aimed to establish a baseline comparison of Arc levels in the DG across the sleep and wake periods in naïve Fischer (CDF) rats. Specifically, we quantified Arc immunoreactivity at four different timepoints across the light/dark cycle, namely 3 h and 8 h into each the light period and dark period. By analyzing video recordings of home cage activity, we first confirmed that rats were predominantly sleeping or awake, respectively, during the last three hours prior to sacrifice ( Figure 1A ). We then carried out immunohistochemistry on hippocampal tissue ( Figure 1B ) and computed cell density of immunopositive Arc cells in the DG. We found no statistically significant difference across the four time points (F _(3, 40)_ = 1.425, p = 0.25; Figure 1C ), demonstrating that DG granule cell activation remains stable across the sleep-wake period under naïve conditions.

An earlier study showed that REM sleep recovery following a period of REM sleep deprivation was associated with a dramatic rise in Arc⁺ DG cells compared to control animals. Interestingly, Arc expression remained comparatively stable in the CA1 and CA3 hippocampal subregions. Across whole hippocampus, Arc mRNA expression was positively correlated with REM sleep quantities across control animals, REM-restricted rats, and REM sleep recovery rats (Renouard et al., 2015). Another study found that post-learning sleep increases in REM sleep P‑wave density (pontine bursts during REM sleep), which was positively correlated with Arc protein levels in the dorsal hippocampus (Ulloor & Datta, 2005). REM-induced Arc expression was also demonstrated in an *in vitro* paradigm mimicking intermittent REM sleep epochs, where Arc protein expression reached levels even exceeding levels following chronic stimulation (Soulé et al., 2012). Alongside our finding that Arc⁺ cell density remains stable across light–dark periods under baseline conditions, these studies suggest that Arc expression during sleep may be selectively driven by either learning- and memory-related behavioral engagement or increased sleep debt. Indeed, another study showed that REM-induced Arc expression occurs specifically in animals that were exposed to novel objects during the preceding wake period (Calais et al., 2015). A limitation of the current study is the inability to distinguish REM sleep behaviorally, limiting our ability to relate the observed Arc levels to REM sleep specifically.

In summary, the primary finding of this study is that Arc⁺ DG cell density remains stable across sleep–wake cycle under baseline conditions. However, when considered in the context of prior work examining Arc expression during sleep, these results underscore the need for future studies to take careful consideration of regional specificity, the distinct contributions of REM and NREM sleep, and the influence of behavioral experiences during prior wake. Moreover, given the highly dynamic temporal profile of Arc expression (Ramirez-Amaya et al., 2013; Vazdarjanova et al., 2006), the timing of these experiences relative to sleep is likely to be a critical determinant of observed effects.

## Methods

**Animals**

Fischer (CDF) rats (F344/DuCrl, RRID:RGD_734478) (n=44; ~12 weeks old; 50:50 male:female) were used in this study. Rats were housed in pairs, fed *ad libitum* , and kept in an environment with a controlled 12-hour light/dark cycle. The light-dark cycle was set such that lights were turned on at 7:00 AM, corresponding to zeitgeber time 0 (ZT0). All animal procedures were approved by the Institutional Animal Care and Use Committee of Loyola University Maryland.

**Sleep/wake estimation**

Behavioral activity was scored continuously throughout the 3-h video recordings using BORIS software. Wakefulness was defined as visible movement and/or open eyes, whereas sleep was defined as immobility and closed eyes. The videos were continuously monitored during scoring, allowing the scorer to maintain identification of each individual rat throughout the recording despite pair housing. Accordingly, each video file was analyzed separately for each rat, resulting in two independent scoring passes per video file.

**Tissue collection**

Perfusion and collection of the rodent brains were performed at four different timepoints, namely 3 h into the light (sleep) period (ZT3), 8 h into the light (sleep) period (ZT8), 3 h into the dark (active) period (ZT15), and 8 h into the dark (active) period (ZT20). Rats were anesthetized with 5% isoflurane for ~3 min in a chamber and maintained with 2% isoflurane through a nose cone. Animals were perfused transcardially at a rate of 35 mL/min with cold PBS for 2 min and then with cold 4% paraformaldehyde (PFA) in PBS (pH 7.2–7.4) for 13 min. Brains were removed and placed in cold 4% PFA in PBS (pH 7.2–7.4) to postfix at 4°C overnight. Brains were incubated in 10% glycerol in 0.1M phosphate buffer at 4°C for 24 h, followed by 20% glycerol in 0.1M phosphate buffer at 4°C for 24 h, cut laterally to separate the left and right hemispheres, and then frozen in chilled 2-methylbutane. A sliding microtome was used to make coronal sections at a nominal thickness of 30 µm. Sections were stored in tissue cryoprotectant solution (TCS) at –80 °C until further processed. 

**Immunohistochemistry**

Sections were washed 3× with TBS and then incubated in 1% H _2_ O _2_ for 15 min. Sections were washed again 3× in TBS and then placed in blocking buffer (5% normal horse serum with 0.3% Triton-X) for 1 h at room temperature on a shaker. Arc antibody was diluted in blocking buffer (1:1000), added to sections, and incubated at 4°C for two days on a shaker. Sections were washed 3× with TBS and then incubated with secondary antibody (Vector Laboratories, BA-1100; 1:1000) diluted in TBS and incubated for 1.5 h at room temperature on a shaker. Sections were washed 3× with TBS and then incubated for 1 h in avidin–biotin complex (ABC Elite kit, Vector Laboratories, PK-6100) for 1 h. Immunoreactivity was visualized using diaminobenzidine (DAB) with nickel enhancement (Vector Laboratories, SK-4100). Sections were mounted, air-dried overnight, dehydrated, and coverslipped with DPX mounting medium (Electron Microscopy Sciences).

**Cell quantification and analysis**

Arc-positive neurons in the dorsal DG (AP: −3.12 to +5.88 mm relative to bregma) were counted manually under 10× magnification using a compound microscope. Inter-rater reliability was assessed for both Arc-positive neuron counts and DG area measurements using percent agreement, calculated as ((Number of Agreements)/(Total Number Rating) ×100). Inter-rater reliability was considered acceptable when the percent agreement exceeded 75%. If the initial paired measurements were outside the acceptable range, both raters independently repeated the measurement one additional time. A Zeiss Axiolab 5 microscope was used to image each hippocampus 5× magnification, and ImageJ was used to outline the granule cell layer of the DG. To account for variability in the thickness between sections, a confocal microscope was used to measure thickness of each section. Cell counts, area, and thickness were then used compute cell density. Final values were based on 9.18±2.9 (mean±SD) sections/animal. Statistical analysis was performed using GraphPad Prism (version 11.0.0), where groups were compared using an ordinary one-way ANOVA. 

## Reagents

**Table:** 

**ANTIBODY**	**ANIMAL AND CLONALITY**	**AVAILABLE FROM**
anti-Arc	Rabbit polyclonal	Synaptic Systems 156 003
